# Cost-effectiveness analysis of rituximab versus natalizumab in patients with relapsing remitting multiple sclerosis

**DOI:** 10.1186/s12913-022-07495-4

**Published:** 2022-01-28

**Authors:** Mehdi Rezaee, Mohammad Hossein Morowvat, Maryam Poursadeghfard, Armin Radgoudarzi, Khosro Keshavarz

**Affiliations:** 1 Department of Health Management, Policy and Economics, Faculty of Management and Medical Information Sciences, Kerman University of Medical Sciences, Kerman, Iran; 2 Pharmaceutical Sciences Research Center, Shiraz University of Medical Sciences, Shiraz, Iran; 3 Department of Pharmaceutical Biotechnology, School of Pharmacy, Shiraz University of Medical Sciences, Shiraz, Iran; 4 Clinical Neurology Research Center, Shiraz University of Medical Sciences, Shiraz, Iran; 5 Health Human Resources Research Center, School of Health Management and Information Sciences, Shiraz University of Medical Sciences, Shiraz, Iran; 6 Emergency Medicine Research Center, Shiraz University of Medical Sciences, Shiraz, Iran

**Keywords:** Multiple Sclerosis, Natalizumab, Rituximab, QALY

## Abstract

**Introduction:**

Multiple sclerosis (MS) is an inflammatory disease in which the myelin sheaths of the nerve cells in the brain and spinal cord, which are responsible for communication, are destroyed and cause physical signs and symptoms. According to studies, anti-CD20 monoclonal antibodies have significant results in the treatment of this disease. Thus, the aim of the present study was to determine the cost-effectiveness of rituximab against natalizumab in the patients with RRMS in southern Iran in 2020.

**Methods:**

This is an economic evaluation including cost-effectiveness analysis in which the Markov model with a lifetime horizon was used. The study sample consisted of 120 patients randomly selected from among those referred to the MS Association and the Special Diseases Unit of Shiraz University of Medical Sciences. In this study, the costs were collected from a societal perspective, and the outcomes were obtained in the form of Quality Adjusted Life Years (QALY) and the mean relapse rate. The TreeAge pro 2020 and Excel 2016 software were used for data analysis.

**Results:**

The comparative study of rituximab and natalizumab showed that the patients receiving rituximab had lower costs ($ 58,307.93 vs. $ 354,174.85) and more QALYs (7.77 vs. 7.65). In addition, the incidence of relapse by rituximab was lower compared to natalizumab (1.15 vs. 2.57). The probabilistic one-way sensitivity analysis showed the robustness of the results. The scatter plots also showed that rituximab was more cost-effective for the patients in 100% of the simulations for the threshold of < $ 37,641.

**Discussion and conclusion:**

According to the results of this study, rituximab had higher cost-effectiveness than natalizumab. Therefore, it could be a priority for RRMS patients compared to natalizumab because it reduced treatment costs and increased effectiveness.

## Background

Multiple sclerosis (MS) is an inflammatory disease in which the myelin sheaths of the nerve cells in the brain and spinal cord are damaged. The damage can impair the ability of some parts of the nervous system responsible for communication and cause many physical signs and symptoms [1, 2]. Although the cause of the disease is unknown, its main mechanism is damage by the immune system or disruption of myelin sheath-producing cells [2]. The number of MS patients has increased from 2.3 million in 2013 to 2.8 million in 2020; however, the incidence rates vary markedly in different parts of the world and in different communities [3]. The disease usually occurs at the age of 20-50 and is about 3 times more common among women than men [4]. The prevalence of MS in Iran has also increased significantly in recent years, reaching from 73.7 per 100,000 in 2006 to 137.6 per 100,000 in 2018 [5]. According to the studies carried out from 2011 to 2020 in different provinces, the prevalence of the disease in Iran ranged from 27.7 per 100,000 in East Azerbaijan to 148.06 per 100,000 in Tehran [6–12]. In addition, a meta-analysis research carried out in 2020 indicated that the annual prevalence of MS had increased by 2.3% during 1985-2018 [13].

According to studies, MS has a significant negative effect on patients’ quality of life [14]. The average life expectancy of the patients is 40 years from the onset of the disease, which is 5 to 10 years less than the average life expectancy of non-infected people. About 60% of MS patients reach the age of 70 [15]. Furthermore, studying the patients with reduced disability Disease-modifying therapies (DMT) showed that about 90% of the total cost of the patients with mild to moderate disability was associated to drugs, and it was found out that the costs of drugs (except DMT) and non-medical sources were higher for the patients with a more severe disease. The increased disability also had a significant effect on health-related quality of life and the fatigue in daily life [16].

Published studies have also shown that despite the high prevalence of MS among young people (mean age of onset < 30), the prevalence of the disease is now increasing among the people over 40 years of age, with 21.3% of relapsing remitting multiple sclerosis (RRMS) patients suffering from late-onset relapsing-remitting multiple sclerosis (LORRMS) with a mean onset age of 47.8 years [17]. Although the number of Late- onset multiple sclerosis (LOMS) women is still higher than men, the increase trend is greater in the male population as their ages increase [18]. In addition, late-onset RRMS men reach severe disability faster than young RRMS ones [19].

Furthermore, as age increases and underlying diseases appear in the patients with MS, the prevalence of polypharmacy (use of multiple drugs) becomes more common in older RRMS patients. It was shown in a study that 28.6% of elderly patients used multiple drugs [20]. thus, a balance should be made between the risks of drug use and effectiveness in patients with LORRMS due to population aging and the presence of underlying diseases as well as the prevalence of polypharmacy [21]. Studies have also shown that patients who frequently changed their DMT were at an increased risk of cancer because these drugs could eventually alter the immune system and indirectly increase the potential for cancer [22].

Studies show that MS imposes a significant economic burden on patients and societies. The findings of a study indicated that the cost of annual health care per MS patient increased from $ 45,471 in 2011 to $ 62,500 in 2015, representing an average annual growth of 8.3%. In addition, the annual cost of purchasing medication for each MS patient increased from $ 26,772 to $ 43,606, with an average annual growth rate of 13.0% [23]. A study conducted in Spain also found that the total cost of MS was € 1395 million per year, with an average annual cost of € 30,050 per patient. In addition to the costs, the disease had a significant effect on patients’ quality of life, and it was estimated that sclerosis imposes would cause a loss of 13,000 quality adjusted life years (QALYs) annually. In general, MS had a great economic impact on the Spanish society and a significant effect on the patients’ quality of life [24].

There is currently no definitive cure for MS, but various drugs are being used to better control the disease and better adapt the patients to the conditions, amongst which are interferon beta and glatiramer acetate, oral drugs (dimethyl fumarate, teriflunomide and fingolimod) as well as natalizumab and alemtuzumab [25]. Monoclonal antibodies are currently very popular with specialists. They act against the CD20 protein like rituximab, ocrelizumab, and ofatumumab that have all shown positive results and are being studied as potential drugs [26]. rituximab is a drug used to treat some types of autoimmune diseases and cancers. It is a chimeric monoclonal antibody against the CD20 protein that is commonly found on the surface of B-lymphocytes. Rituximab binds to CD20 and causes cell death. Relatively common complications that often occur 2 h after starting the injection include skin rash, itching, low blood pressure, and dyspnea [27].

DMTs for MS in Iran include injectable drugs (β-interferons and Glatiramer acetate), oral drugs (fingolimod, Teriflunomide, and Dimethyl fumarate), and infusion drugs (natalizumab and Ocrelizumab) [28], and physicians use first-line drugs (beta interferons, glatiramer acetate, Teriflunomide, and dimethyl fumarate) for the treatment of MS [28], depending on the patients’ conditions and evaluation of the disease. In case of insufficient response to these drugs, physicians prescribe second-line drugs (fingolimod and natalizumab) [28], but given that the use of natalizumab has a very high risk of developing Progressive Multifocal Leukoencephalopathy (PML) in the patients, studies on the treatment options after discontinuation of natalizumab showed that although the drugs that targeted CD20 + B cells, such as rituximab and ocrelizumab, were not significantly associated with PML risk, they were very effective in suppressing inflammatory activity in RRMS [29]. Also, even if discontinuation of natalizumab leads to relapse of MS in patients at high risk for PML, suspension of treatment and transfer to other highly effective DMTs is a possible strategy to limit the occurrence of PML [30]. In addition, a 2020 study evaluating the effectiveness of rituximab in patients with RRMS who had an inadequate response to DMTs found that after 18 months of treatment with rituximab, the relapse rate decreased significantly, but the mean EDSS remained almost unchanged. Besides, infusion-related side effects occurred in 60% of the patients in the first infusion but most of them were mild [31].

In general, considering the increased number of MS patients as well as the increasing costs of the disease imposed on the patients and societies [32], and since the researchers found no study conducted to evaluate the cost-effectiveness of rituximab and natalizumab, the present research aimed to determine the cost-effectiveness of these two drugs in the patients with RRMS in Fars province in 2020 in order to identify the most cost-effective one for the patients with RRMS and to help managers, policy makers, and specialists prescribe better drugs and use the limited resources properly.

## Methods

### Overview and model structure

This is a full economic evaluation of cost-effectiveness analysis type conducted on the patients using natalizumab (300 mg 1 injection per months) and rituximab (1000 mg 1 injection per 6 months) who referred to the MS Association and the Special Diseases Unit of Shiraz University of Medical Sciences in 2020. According to the results of the pilot study and considering α = 5%, SD = 1.01, and d = 0.25, the sample size was determined to be 60 in each group. The patients were selected through the random sampling method and entered the study. The inclusion criteria were the use of the mentioned drugs for at least 1 year and the willingness to participate in the research.

**Fig. 1:**
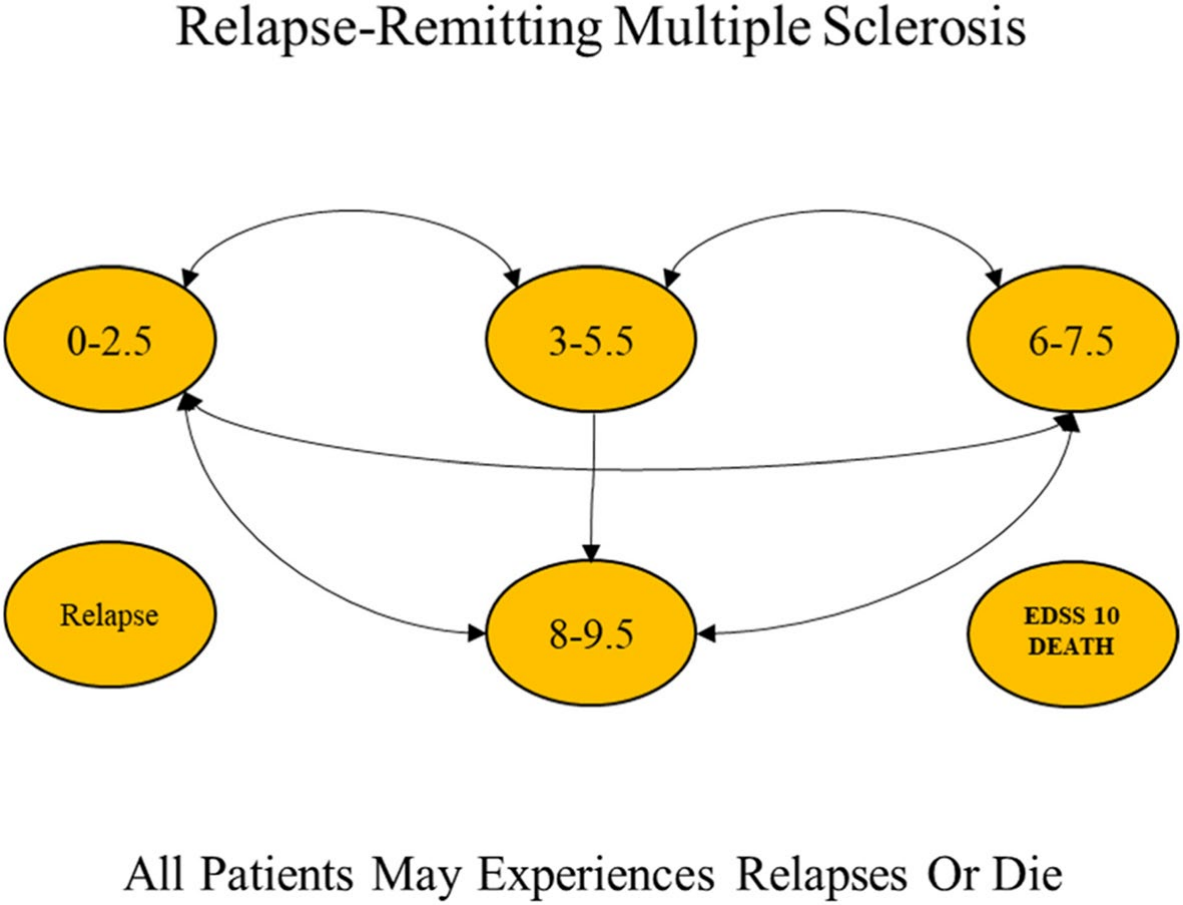
Schematic diagram of Markov model for RRMS [34]

Due to the chronic and relapsing nature of MS, the Markov model was used in this study. Figure 1 indicates a schematic diagram of the Markov model for the disease, The time horizon in this study was the lifetime and the interval of the annual Markov cycles. At the time of entering the model, the patients had an EDSS of 0-2.5; they were mostly female (74%) with a mean age of 34 years. For each one-year cycle in the model, the patients remained in their current states or were transferred to other ones, and any patient might experience relapse or death [33, 34]. As long as the patients are not transferred to a higher level than EDSS 3–5.5, they remain in RRMS status, and when transited to the EDSS 6–7.5 level or higher, they are considered as Secondary Progressive Multiple Sclerosis (SPMS) and their DMTs (rituximab and natalizumab) are stopped [35]. All the probabilities of transition to other states, Annualized Relapse Rate, and the probability of death in each Expanded Disability Status Scale (EDSS) were extracted based on previously published studies and were presented in Table 1 [36, 39–41]. It should be noted that some clinical trial data were reported as rates. They were first converted to transition probabilities using the following formula, and the transition probabilities were then entered the model [42].

$$\mathrm P=1-\exp\left(-\mathrm{rt}\right)$$

where p is wanted transition probability and r is the overall rate over the time of t. Since the time horizon was over one year, the cost data and the outcomes of the model were discounted based on the discount rates of 5.8% [43] and 3% [44], respectively.

**Table 1:** Annual transition probabilities (%) for EDSS states and annual probability of relapse (%) in patients with RRMS

EDSS From\To	0-2.5	3-5.5	6-7.5	8-9.5	source	Death	Source
0-2.5	82.567	15.93	1.47	0.033	[36, 37, 38 ]	1.343	[39]
3-5.5	10.07	59.73	28.8	1.4		1.718	
6-7.5	0.3	5.5	81.95	12.25		2.685	
8-9.5	0	0.1	4.15	95.75		5.450	
annual probability of relapse		Mean			Source		
Rituximab		0.104			[40]		
Natalizumab		0.265			[41]		

The outcomes entered this model included QALYs and the costs of any health condition and any treatment strategy. Utility scores were extracted using the EQ-5D-3L questionnaire, and the health outcomes were valued based on QALY [45]. To measure the utility scores, face-to-face interviews or telephone calls with 120 MS patients were done in 2020. The interviews were conducted with the outpatients referred to the hospitals and clinics affiliated to Shiraz University of Medical Sciences. It is worth noting that the EQ-5D-3L questionnaire is a standard tool used to measure health outcomes and includes 5 questions that measure mobility, self-care, usual activities, pain / discomfort, and anxiety / depression. Respondents can score each aspect from 0 to 1, and higher scores mean better utility. The questionnaire was introduced by the EuroQol group in 1990 (https://euroqol.org/). The MS patients who were willing to participate in the present study were interviewed accordingly. Once the EQ-5D-3L questionnaire was completed, its 5-digit codes were changed into numerical utility by considering the values of Iran determined by Goodarzi et al. [46] using the time trade-off method (TTO).

The community perspective was also used to extract the costs. The relevant costs from a community perspective included Direct Medical Costs (DMC), Direct Non-Medical Costs (DNMC), and Indirect Costs (IC). The DMC of each drug was collected retrospectively from March 21, 2019, to March 20, 2020, using a researcher-made checklist and by referring to specialists’ offices physicians and the MS Association of Fars Province. The DMC included the costs of visits, purchase of medicine, Magnetic Resonance Imaging (MRI), laboratory tests, hospitalization, and physiotherapy, where the unit price of each item and the rates of their use per year are reported in Table 2. It is worth noting that the unit price of each item is based on the annual tariff announced by the Ministry of Health and Medical Education [47]. Furthermore, the rate of using each service was calculated based on the experts’ opinion.

**Table 2:** The rate of patients’ annual use of each of the direct medical costs items and the unit price of each of them in 2019-2020

Cost components	natalizumab		rituximab	
	Count	Unit Price (PPP $)	Count	Unit Price (PPP $)
Physicians’ Visits	6	44	2	44
Main Medicines	12	2745	2	1019
Complete Blood Counts	2	30	2	30
Biochemistry Tests	2	30	2	30
Urinalysis Tests with Microscopy	–	–	2	25
Tuberculin Skin Test	–	–	1	25
JC Virus Test	2	73	–	–
Serology (HIV, HBV and HCV) Test	–	–	1	25
Magnetic Resonance Imaging (MRI)	1	500	3	500
Physiotherapy & Other Services Costs	They vary based on the patients’ conditions			
Hospitalization	They vary based on the patients’ conditions			
Supplementary Medicines	They vary based on the patients’ conditions			

The DNMC included the costs of traveling to other cities and accommodation as well as the meals consumed by the patient and his/ her family, and the nursing home care expenses determined by asking the patients. The human capital approach was used for IC calculation [48–50].

In this study, all the expenses were converted into dollars Purchasing Power Parity (PPP) using the exchange rate of each PPP dollar equal to 22,075 Rials in 2019 [51].

### Effectiveness

The mean relapse rate was used to measure effectiveness. To this end, the total number of relapses of all patients was calculated and divided by the total number of years of drug use to obtain the annual relapse rate of the patients. The obtained value was then divided by the number of patients using each drug in order to obtain the mean relapse rate of each drug [52, 53].

### Determining the incremental cost-effectiveness ratio

After the calculation of the costs and utilities in the previous stages, the incremental cost-utility was calculated using the following formula. To make the final decision on the cost-effectiveness of each intended drug, the ICER level was compared with the threshold.

$$\boldsymbol I\boldsymbol C\boldsymbol E\boldsymbol R\boldsymbol =\frac{\mathit C\mathit o\mathit s\mathit t\mathit s\mathit\;\mathit o\mathit f\mathit\;\mathit r\mathit i\mathit t\mathit u\mathit x\mathit i\mathit m\mathit a\mathit b\mathit-\mathit C\mathit o\mathit s\mathit t\mathit s\mathit\;\mathit o\mathit f\mathit\;\mathit n\mathit a\mathit t\mathit a\mathit l\mathit i\mathit z\mathit u\mathit m\mathit a\mathit b}{\mathit O\mathit u\mathit t\mathit c\mathit o\mathit m\mathit e\mathit s\mathit\;\mathit o\mathit f\mathit\;\mathit r\mathit i\mathit t\mathit u\mathit x\mathit i\mathit m\mathit a\mathit b\mathit-\mathit\;\mathit O\mathit u\mathit t\mathit c\mathit o\mathit m\mathit e\mathit s\mathit\;\mathit o\mathit f\mathit\;\mathit n\mathit a\mathit t\mathit a\mathit l\mathit i\mathit z\mathit u\mathit m\mathit a\mathit b}$$

Due to the lack of an explicit threshold for Willingness To Pay (WTP) in Iran, the WHO’s proposal for developing countries was used, i.e. the willingness to pay per QALY was one to three times the per capita Gross Domestic Product (GDP) [54]. In Iran, GDP was $ 12,547 in 2019 [55] based on which the threshold for willingness to pay was $ 37,641 (3* GDP). The Excel 2016 and TreeAge Pro 2020 software were also used for data analysis. Accordingly, TreeAge software was used to analyze the Markov model and Excel software was used to collect and summarize cost data, efficacy, and individual patient data analysis.

### Sensitivity analysis

The researchers used the one-way sensitivity analysis and Probabilistic Sensitivity Analysis (PSA) to investigate the effects of parameter uncertainty on the results. In order to perform the one-way sensitivity analysis, some key parameters such as cost and utility were changed by 20% for each drug strategy and the results were presented in the form of a Tornado Diagram. In addition, since the utility and cost variables in the present study were measurable and probabilistic, PSA was performed and they were considered as distributions, so that beta distribution (ß) was used to determine the distribution of utility values (ranged from 0 to 1) and gamma distribution was used to determine the cost distribution. Accordingly, Second-order Monte Carlo simulation was performed using 5000 trials. The PSA results are presented using the cost-effectiveness acceptability curve and incremental cost-effectiveness scatter plot. Cost-effectiveness acceptability curve is one of the best curves for planning and policy-making that can help health policy makers and planners identify the probability of cost-effectiveness of each intervention in exchange for different WTPs. On the other hand, scatter plots provide more detailed information in individual comparisons. The plots actually indicate the percentage of the points in the acceptance area, i.e. below the threshold [56].

## Results

The findings of the present research showed that most of the patients were female (74.17%) and housewives (52.50%), and all of the patients had an insurance coverage. In addition, the mean ages of the patients treated with natalizumab and rituximab were 33.4 ± 7.27 and 34.92 ± 5.94 years, respectively.

Tables 3 and 4 show the mean cost, utility, and relapse in the MS patients using natalizumab and rituximab. According to Table 3, the highest mean DMC and DNMC rates were associated to the patients taking natalizumab ($ 34,912.80 and $ 665.70, respectively). Also, the cost of purchasing the main drug was the highest type of DMC in natalizumab and rituximab groups ($ 32,942.24 for natalizumab and $ 2038.51 for rituximab). Furthermore, travel expenses and income lost of the outpatients accounted for the highest DNMCs and ICs in both groups ($ 441.72 and $ 282.83 for natalizumab, and $ 27.94 and $ 254.72 for rituximab, respectively).

**Table 3:** The mean annual costs in RRMS patients using natalizumab and rituximab

Costs	natalizumab		rituximab	
	PPP $	Percentage	PPP $	Percentage
Direct Medical Costs				
Physicians’ Visits	258.00	0.74	89.90	1.82
Main Medicines	32,942.24	94.36	2,038.51	41.33
Supplementary Medicines	695.69	1.99	421.64	8.55
Laboratory Tests	267.18	0.77	220.63	4.47
MRIs	508.09	1.46	1,457.97	29.56
Physiotherapy & Other Services Costs	0.00	0.00	647.27	13.12
Hospitalization & Surgeries	241.60	0.69	56.04	1.14
Total	34,912.80	96.82	4,931.96	91.34
Direct Non-Medical Costs				
Transportation	441.72	66.35	27.94	67.94
Accommodation	53.14	7.98	0.00	0.00
Meals	53.14	7.98	0.00	0.00
Purchasing Auxiliary Tools	117.70	17.68	13.18	32.06
Total	665.70	1.85	41.12	0.76
Indirect Costs				
Income Lost due to Outpatient Visits	282.83	58.97	254.72	59.76
Income Lost due to Hospitalization	79.27	16.53	64.40	15.11
Patient’s Family Costs	117.48	24.50	107.08	25.13
Total	479.58	1.33	426.20	7.89
Total Costs	36,058.08	100.00	5,399.28	100.00

**Table 4:** The means of utility scores and relapses avoided rates in RRMS patients using natalizumab and rituximab

Parameters	natalizumab		rituximab	
	Mean	SD	Mean	SD
Utility Scores				
EDSS 0.0–2.5	0.754	0.186	0.833	0.125
EDSS 3.0–5.5	0.530	0.120	0.621	0.097
Relapse Rates				
EDSS 0.0–2.5	1.024	1.506	0.313	0.528
EDSS 3.0–5.5	1.667	1.782	1.727	0.905

As shown in Table 4, the highest utility score obtained from the EQ-5D questionnaire was that of the RRMS patients using rituximab and with the EDSS of 0-2.5 (0.833 ± 0.125). The lowest mean relapse was that of the patients using rituximab with the EDSS of 0-2.5 (0.313 ± 0.528).

**Fig. 2:**
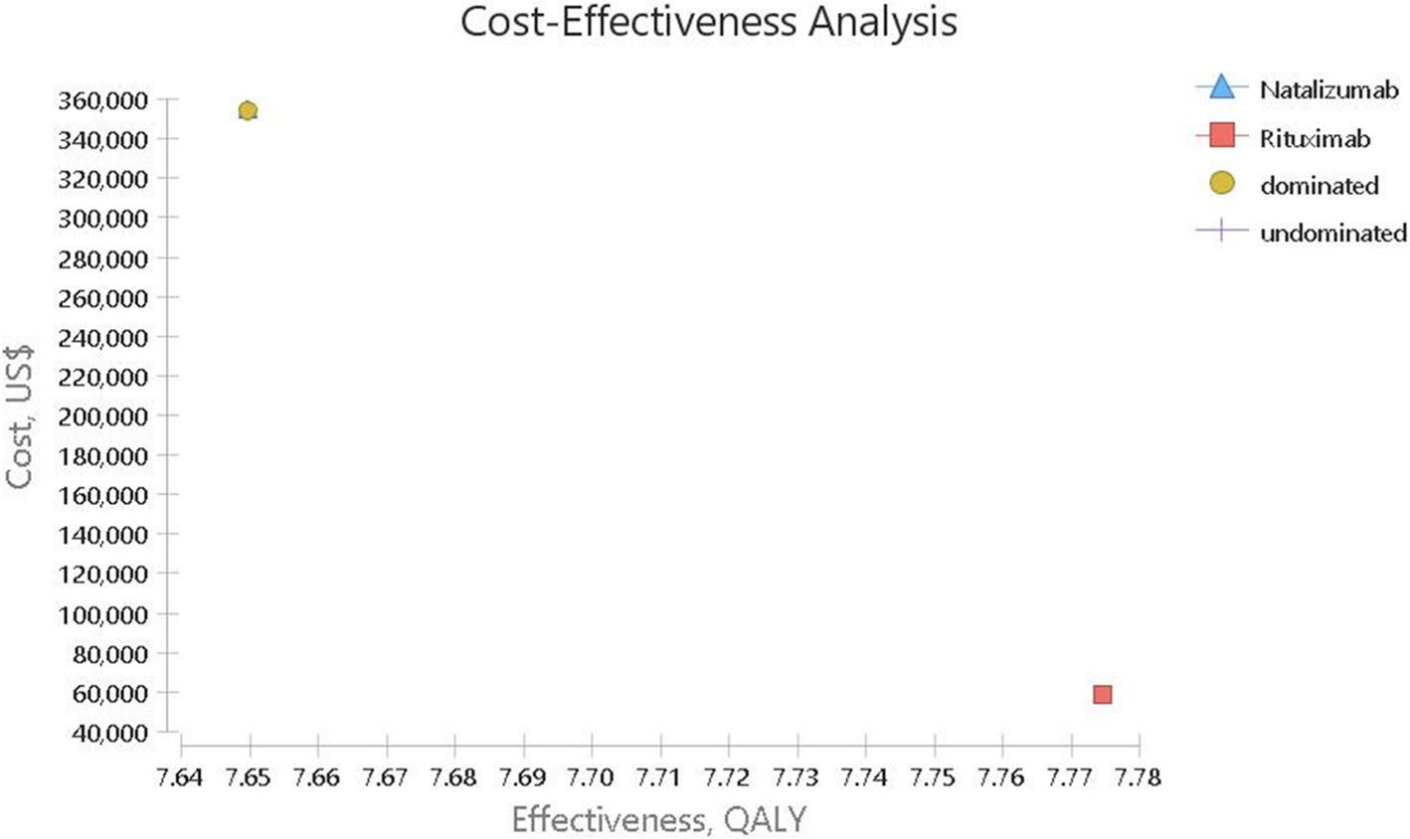
Cost-effectiveness analyses for RRMS patients under treatment with Natalizumab and Rituximab

As presented in Fig. 2 and Table 5, the results of the cost-utility analysis using the Markov model suggested that the mean cost was $ 58,307.93 in the rituximab arm and the QALY was 7.77 as well. However, the mean predicted cost in the natalizumab arm was $ 354,174.85 and the obtained QALY was 7.65.

**Table 5:** The base-case analysis results (the lifetime analysis)

	Strategy	cost (PPP$)	QALY	Incremental Cost	Incremental QALY	ICUR (Incremental cost per QALY Gained) PPP$
Cost-Utility analysis	Natalizumab	354,174.85	7.65	0	0	–
	rituximab	58,307.93	7.77	− 295,867	0.125	dominant

*ICER* Incremental Cost-Effectiveness Ratio, *QALY* Quality Adjusted Life Year

### One-way sensitivity analysis

**Fig. 3:**
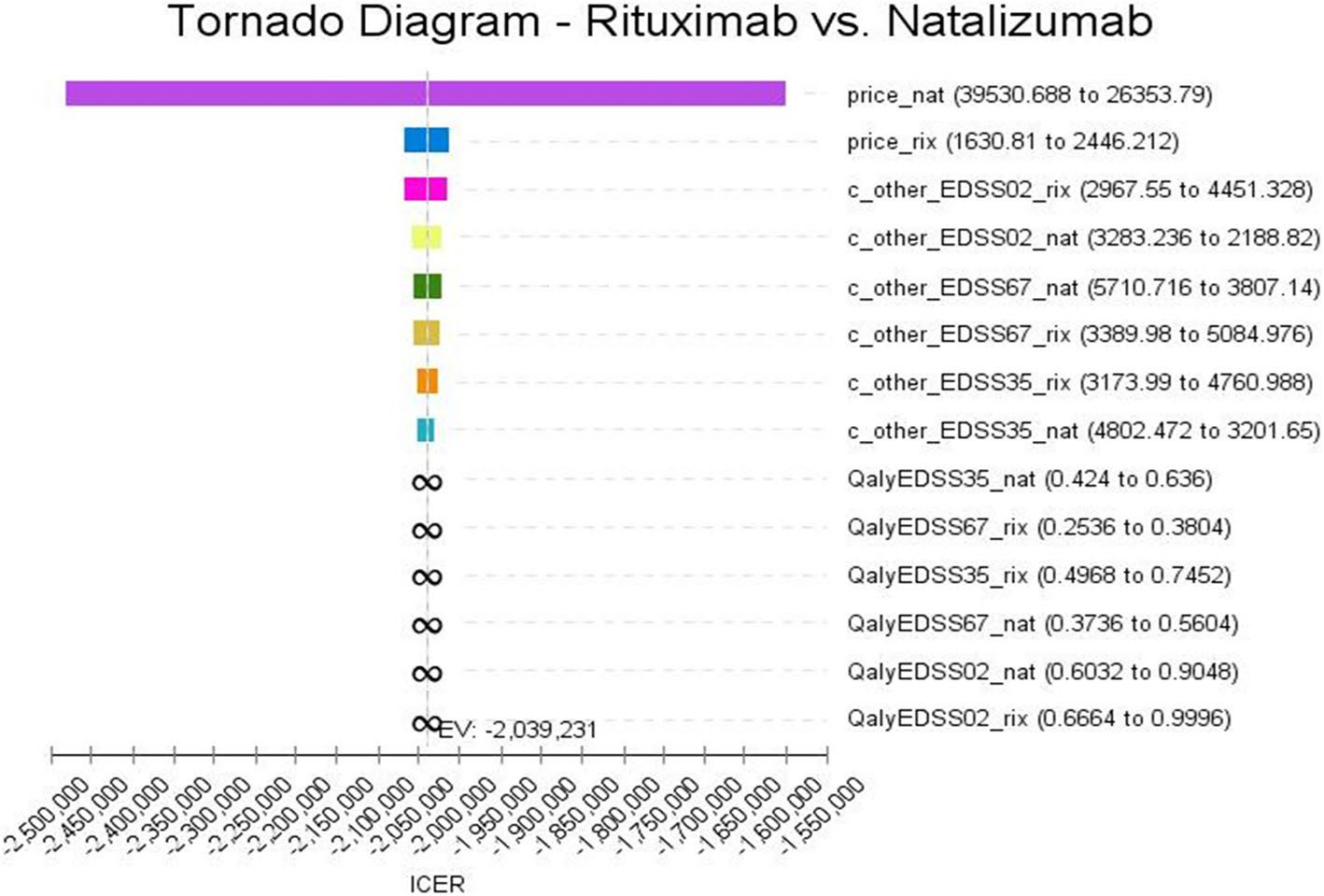
Tornado diagrams of cost-utility of the studied RRMS patients under treatment with Natalizumab and Rituximab

According to the tornado diagram in Fig. 3, ICER was most sensitive to the price of natalizumab and less sensitive to that of rituximab. In addition, the tornado diagram shows that changes in input parameters had little effect on the result and the ICER rate remained negative.

### Probabilistic Sensitivity Analysis (PSA)

**Fig. 4:**
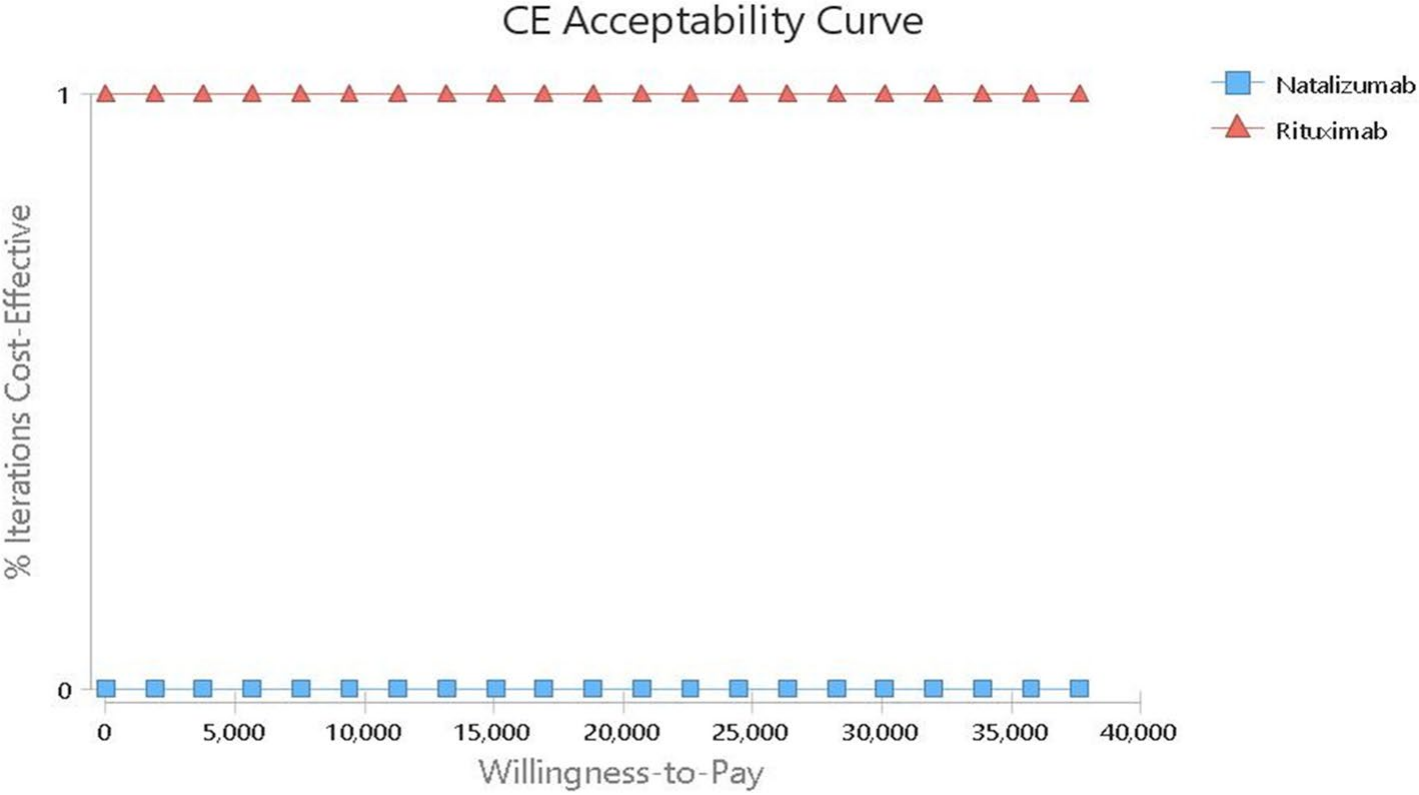
The cost-effectiveness acceptability curves of Rituximab versus Natalizumab based on the QALY obtained through the Monte Carlo simulation

The results of uncertainty measurement are presented using the cost-effectiveness acceptability curves and ICER scatter plot as follows. The acceptability curves indicated that rituximab was the most cost-effective treatment in 100% of simulations for the thresholds lower than $ 37,641 (Fig. 4).

**Fig. 5:**
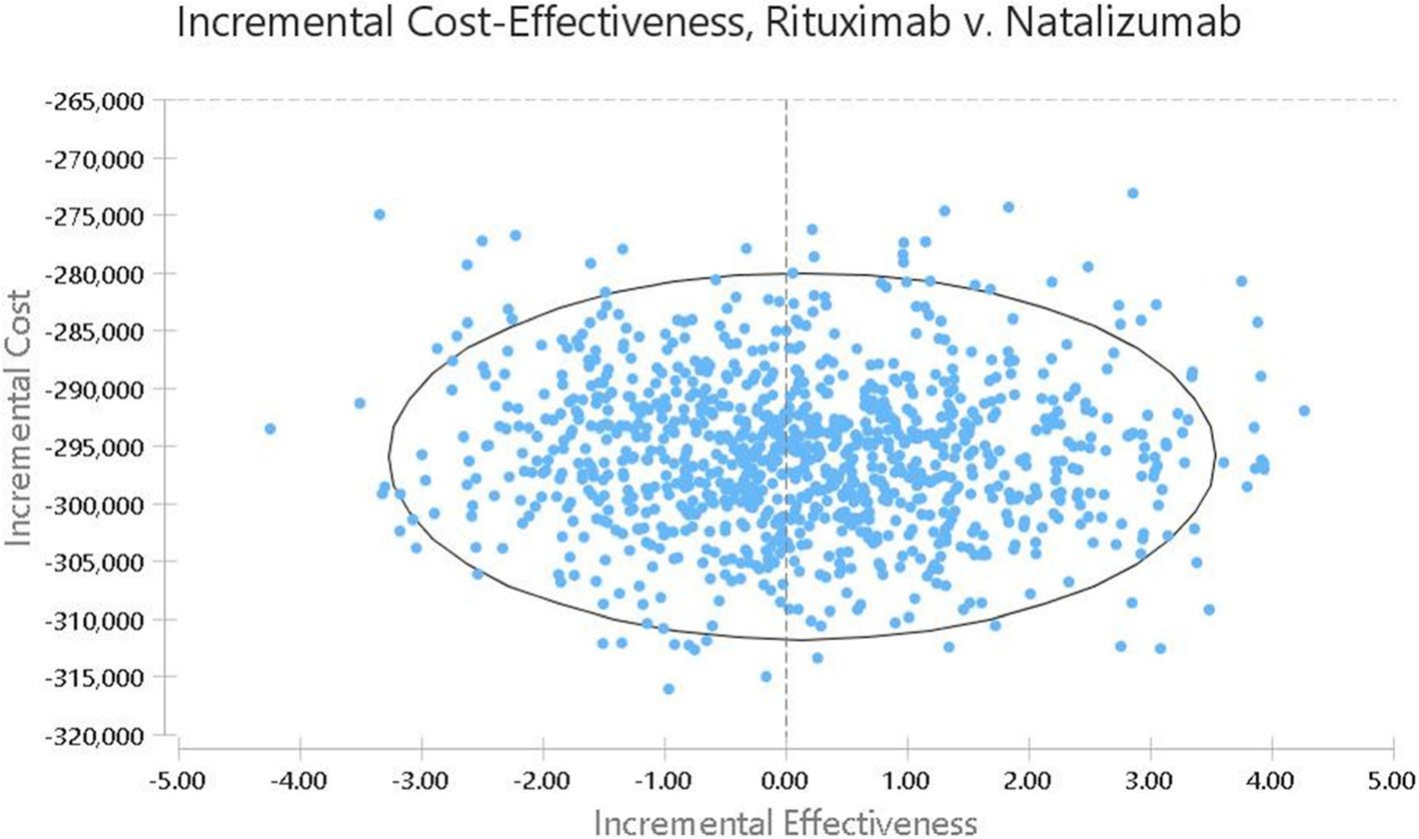
The incremental cost-effectiveness scatter plots of Rituximab versus Natalizumab based on the QALY

The results on the scatter plot showed that rituximab was more effective and less costly in 51.24% of the cases. In addition, although it was less effective in 48.76% of cases, it had a much lower cost, and the ICER level was below the threshold. Overall, rituximab was in the acceptance area and below the threshold in 100% of the cases and defeated natalizumab. so it was a more cost-effective strategy. On the other hand, natalizumab was in the rejection area in 100% of the cases and above the threshold compared to rituximab, and was considered as a non-cost-effective (inefficient) strategy (Fig. 5) .

## Discussion

Along with the identification and approval of new drugs for the management of MS, there has been ever-increasing costs of treating the disease imposed on families and communities [57]. Therefore, conducting economic evaluations seems necessary to identify the cheapest, most effective, and thus most cost-effective medicines and suggest them to doctors for prescription. Such studies can also help health policy makers to reduce patients’ out-of-pocket payments. To the knowledge of the researchers, this is one of the first health-economic studies conducted to compare rituximab and natalizumab in the treatment of RRMS. In fact, this is the first study carried out in Iran on the cost-effectiveness of rituximab versus natalizumab in RRMS patients. According to the findings of this study, the mean annual costs of treatment with natalizumab and rituximab were $ 36,058.08 and $ 5399.28, respectively. Thus, the mean cost of a course of treatment per patient was lower with rituximab than with natalizumab. The reasons for such a difference could be the higher price of natalizumab compared to rituximab, and the more frequent use of the former drug (12 doses per year) compared to the latter (2 doses per year). In their studies, D’Amico et al. [58] and Bellinvia et al. [59] showed that rituximab was a low-cost option compared to other approved drugs in the treatment of MS. Furthermore, the study by Chisari et al. [26] showed that the annual cost of rituximab was significantly lower than other approved drugs used in the treatment of MS in Europe and USA. Hartung [60] also showed that the annual cost of MS drugs was about $ 70,000, which is consistent with the results of the present study.

The direct medical, direct non-medical, and indirect costs of natalizumab were $ 34,912.80 (96.82% of the total costs), $ 665.70 (1.85% of the total costs), and $ 479.58 (1.33% of the total costs), respectively. However, the costs were 4931.96 (91.34% of the total costs), 41.12 (0.76% of the total costs), and 426.20 (7.89% of the total costs) for rituximab. Direct costs of both drugs accounted for most of the costs, the largest share of which was that of purchasing the main drug (94.36 and 41.33%, respectively). In this regard, the findings of this study are consistent with those of the studies conducted in Iran by Rezaei et al. [61] and Taheri et al. [62], and the ones carried out abroad by Dahham et al. [63], Brodszky et al. [64], Garcia et al. in Panama [65], and Ernstsson et al. [66].

The results of the present research showed that the highest utility rate and the lowest relapse rate in treatment with each drug was found in the patients with an EDSS of 0-2.5, and as disability increased, the life utility rate decreased and the relapse rate increased as well. This might be due to the fact that in higher EDSSs, the disease usually progresses and the patients’ limitations increase; so, it is natural for the relapse rate to get higher and the utility rate to decrease [67, 68]. In this respect, the results of this study are in line with the ones by Torgauten et al. (2021), Hellgren et al. (2020), Boremalm et al. (2019), and Yamout et al. (2018) [40, 69, 70, 71].

Based on the results of the Markov model analysis, the costs of natalizumab and rituximab were respectively $ 354,174.85 and $ 58,307.93, and the obtained QALYs were 7.65 and 7.77 over the lifetime horizon. Therefore, since natalizumab had higher cost and lower QALY, it was considered the dominated option in cost-effectiveness analysis. Thus, rituximab was more cost effective than natalizumab.

Rezaei et al. [61] conducted a study in Iran and examined the cost-utility of natalizumab versus fingolimod. They found out that the mean cost per patient during life was $ 58,751 in the fingolimod arm and the utility was 8.09 QALY, but in the natalizumab arm, the mean cost and the obtained QALY were $ 204,264 and 7.37, respectively. Thus, natalizumab had higher cost and lower QALY and was the dominated option. This is consistent with the results of the present study.

In their study, Taheri et al. [62] examined the cost-effectiveness of alemtuzumab versus natalizumab and concluded that the total discounted costs per patient were $ 147,417 and $ 150,579, respectively. In addition, the mean discounted QALYs were estimated at 7.07 and 6.05 for alemtuzumab and natalizumab, respectively, over a period of 20 years. Therefore, natalizumab was the dominated option, which is consistent with the present study.

Walter et al. [72] also evaluated the cost-utility of alemtuzumab versus interferon beta, fingolimod, and natalizumab for relapsing MS in Austria and found that alemtuzumab was the dominant option due to having higher total QALY (4.88) and lower total cost (€ 137,409) in comparison with interferon beta-1a (€ 200,133 and QALY of 4.38), fingolimod (€ 240,903 and QALY of 4.64), and natalizumab (€ 247,758 and QALY of 4.40). This is in line with the results of the present study.

In the study by Hettle et al.  [37] on the cost-effectiveness of cladribine versus alemtuzumab and natalizumab in England, it was found that cladribine was the dominant strategy over alemtuzumab and natalizumab when compared two by two, and quite dominant in the incremental analysis (i.e. they were cheaper and more effective). The total costs and QALYs discounted for cladribine, alemtuzumab, and natalizumab were 92,484 and QALY 9.450, 104,136 and QALY 8.482, and 212,969 and QALY 7.739, respectively, which is consistent with the present study.

Montgomery et al. [73] in the UK examined the cost-effectiveness of fingolimod and natalizumab and found that the cost of the former was £ 334,897.93 and its obtained QALY was 6.18. However, the cost and QALY of the latter were £ 337,501.15 and 6.35, respectively. The obtained ICER which was below the threshold in the UK (threshold ≤15,313.06) showed that fingolimod was the cost-effective option, and this is consistent with the present study.

The results of the one-way sensitivity analysis indicated that in the cost-utility analysis, the ICER value was negative and the highest sensitivity was to the price of natalizumab, but considering that in the cost-utility sensitivity analysis the ICER value remained negative, the results of the study had required robustness. In addition, the results of probabilistic sensitivity analysis showed that rituximab regimen was more cost-effective than natalizumab and in all cases, it was in the acceptance area and below the threshold. Thus, it had obtained the best results in terms of the mentioned prices. The results also showed that doing sensitivity analysis did not change the status of rituximab as the most effective drug regimen, suggesting the robust study results. In this regard, the present study is in line with those of Rezaei et al. (2019) and Taheri et al. (2019) in the country, and Walter et al. (2018), Hettle et al. (2018), and Montgomery et al. (2017) abroad [37, 61, 62, 72, 73].

One limitation of the present study was the self-reporting of the patients or their companions about direct non-medical and indirect costs, as they were likely to forget or approximate some of the costs. In addition, intangible costs were not calculated in this study due to the inability to measure them accurately.

Another limitation of this study is that other comparators were not considered which are commonly used in Iran, such as β-interferons, fingolimod, and ocrelizumab.

## Conclusions

The results of the present research suggested that rituximab had higher cost-effectiveness and cost-utility than natalizumab. Therefore, considering the results of the sensitivity analysis and the robustness of the results, rituximab treatment is suggested as the first priority (compared to natalizumab) to treat the patients with RRMS, and health policy makers and managers should try to increase insurance coverage and reduce the patients’ out-of-pocket payments.

## Data Availability

The datasets generated and analyzed during the current study are not publicly available because they contain information that could compromise the privacy of research participants, but are available from the corresponding author upon reasonable request.

## Abbreviations

MS: Multiple Sclerosis
LOMS: Late- Onset Multiple Sclerosis
RRMS: Relapsing Remitting Multiple Sclerosis
LORRMS: Late- Onset Relapsing-Remitting Multiple Sclerosis
QALY: Quality Adjusted Life Years
DMT: Disease-Modifying Therapies
PML: Progressive Multifocal Leukoencephalopathy
EDSS: Expanded Disability Status Scale
DMC: Direct Medical Costs
DNMC: Direct Non-Medical Costs
IC: Indirect Costs
TTO: Time Trade-Off
PPP: Purchasing Power Parity
GDP: Gross Domestic Product
PSA: Probabilistic Sensitivity Analysis
WTP: Willingness to Pay
